# Combined ultrasound-guided C2 DRG pulsed radiofrequency and suboccipital myofascial plane block for cervicogenic headache: a retrospective study

**DOI:** 10.3389/fpain.2026.1725798

**Published:** 2026-01-26

**Authors:** Dan Li, Xing Jin, Jingyu Li, Meige Li, Shuaichen Jin, Wenzhe Jin

**Affiliations:** 1 Department of Endocrinology, Yanbian University Hospital, Yanji, Jilin, China; 2 Department of Pain, Yanbian University Hospital, Yanji, Jilin, China

**Keywords:** cervicogenic headache, myofascial pain syndromes, radiofrequency ablation, spinal ganglia, ultrasonography

## Abstract

**Background:**

Cervicogenic headache (CEH) is often refractory to monotherapies, and treatment strategies combining neuromodulation and myofascial interventions may offer additional benefits. This study evaluated the clinical effectiveness of ultrasound-guided C2 dorsal root ganglion (DRG) pulsed radiofrequency (PRF) combined with suboccipital myofascial plane block (SMPB) in subjects with CEH.

**Methods:**

This retrospective study analyzed 67 CEH subjects treated with PRF alone (*n* = 28) or combined PRF + SMPB therapy (*n* = 39). Pain intensity (VAS), headache frequency and duration, Short-Form McGill Pain Questionnaire (SF-MPQ), and Neck Disability Index (NDI) were assessed at baseline, 1 week, 1 month, 3 months, and 6 months.

**Results:**

The combined treatment produced significantly greater improvement in pain outcomes. At 3 months, VAS scores were 2.09 ± 1.38 in the PRF + SMPB group vs. 3.55 ± 1.11 in the PRF group (between-group difference Δ = −1.46; 95% CI −2.06 to −0.86; *p* < 0.001). Superior improvements were also observed in headache frequency, headache duration, SF-MPQ, and NDI at multiple timepoints. All subjects completed follow-up (attrition rate 0%), and no complications or minor adverse events were reported.

**Conclusions:**

Ultrasound-guided C2 DRG PRF combined with SMPB demonstrated greater reductions in pain and disability than PRF alone in CEH. These findings provide preliminary, hypothesis-generating evidence supporting the feasibility and clinical utility of this multimodal approach. Prospective randomized trials with longer follow-up are warranted.

## 1 Introduction

Cervicogenic headache, first defined by Sjaastad in 1989, refers to headache secondary to pathology of the upper cervical spine (1). It typically manifests as unilateral occipito-temporal pain with restricted cervical mobility and posture-related discomfort, substantially impairing quality of life (2, 3). Cervicogenic headache (CEH) occurs in approximately 4.1% of the general population (4) and is common among patients with severe headache (5).

Beyond its classification as a secondary headache, growing evidence suggests that CEH shares key pathophysiological features with primary headache disorders, particularly migraine and tension-type headache. Peripheral sensitization of nociceptive afferent pathways has been recognized as a common mechanism across headache phenotypes, contributing to pain amplification and allodynia. In migraine, abnormal cervical and pericranial musculoskeletal dysfunctions and postural alterations may serve as persistent peripheral nociceptive inputs that facilitate central sensitization, while in tension-type headache, sustained activation of pericranial muscles and myofascial structures plays a critical role in symptom generation and chronification (6). Consistently, myofascial trigger points have been frequently identified in both migraine and tension-type headache, supporting the concept that pathological myofascial input represents a shared peripheral driver across headache disorders (7).

Within this broader mechanistic framework, CEH is characterized by a dual contribution of neural and myofascial factors. Degenerative changes of the atlantoaxial complex may generate abnormal C1–C3 afferent input and central sensitization, with the C2 dorsal root ganglion serving as a critical relay (8–10). Concurrently, hypertonicity and inflammatory changes of the suboccipital musculature and associated fascia provide sustained peripheral nociceptive drive via the greater, lesser, and third occipital nerves (11, 12). This convergence of cervical neural sensitization and myofascial dysfunction positions CEH at the intersection of spinal and myofascial headache mechanisms, underscoring the rationale for interventions that simultaneously target both components.

Management of CEH typically begins with conservative approaches, including pharmacotherapy, manual therapy, therapeutic exercise, and physical modalities, and escalates to interventional procedures when treatment response is insufficient (13, 14). Pulsed radiofrequency attenuates nociceptive transmission through neuromodulation (15) and has been widely applied to the C2 dorsal root ganglion (DRG) in CEH (16–20). Compared with fluoroscopic guidance (17, 18), ultrasound guidance allows real-time visualization of relevant neurovascular structures and improved targeting accuracy (19, 20). However, neuromodulation alone does not directly address concomitant myofascial dysfunction, which represents an important and potentially modifiable peripheral pain generator in CEH.

The ultrasound-guided suboccipital myofascial plane block involves deposition of local anesthetic, with or without corticosteroid, between the obliquus capitis inferior and semispinalis capitis fascia and has demonstrated clinical benefit in CEH management (8, 21–23), likely through decompression of occipital nerves and attenuation of aseptic fascial inflammation (25, 26). Despite the increasing use of both techniques, existing studies have largely evaluated Pulsed radiofrequency (PRF) and myofascial plane blocks in isolation, and comparative evidence assessing whether the addition of targeted myofascial intervention confers incremental or more durable benefit beyond C2 DRG PRF alone remains scarce.

Based on our prior experience and safety data using an ultrasound-guided posterior approach to the C2 DRG (27), we conducted a single-center real-world retrospective cohort study comparing pulsed radiofrequency alone with its combination with suboccipital myofascial plane block in cervicogenic headache. We specifically aimed to test the hypothesis that simultaneously targeting cervical neural sensitization (via C2 DRG pulsed radiofrequency) and peripheral myofascial nociceptive input (via suboccipital myofascial plane block SMPB) would result in greater and more durable improvements in pain intensity and headache-related disability than neuromodulation alone, without increasing procedure-related adverse events.

## 2 Study population and methods

### 2.1 Patient selection

This retrospective cohort included subjects with unilateral cervicogenic headache diagnosed in the Department of Pain Medicine, Yanbian University Hospital, between May 2021 and August 2024. Diagnosis was jointly confirmed by two senior pain specialists (≥8 years of clinical experience), both of whom routinely manage headache disorders and were trained in the application of the International Classification of Headache Disorders, 3rd edition (ICHD-3), using a harmonized diagnostic workflow (28, 29), requiring: (i) a causal relationship between headache and cervical pathology; (ii) pain provoked by cervical movement or posture; (iii) restricted cervical range of motion; and (iv) ≥50% relief after a diagnostic C2 block. When diagnostic uncertainty arose, cases were reviewed in multidisciplinary discussion.

All subjects underwent standardized physical and neurological examinations. Suboccipital musculature palpation with pressure pain threshold assessment and cervical mobility measurement were routinely performed. To enhance diagnostic accuracy and cohort homogeneity, all participants underwent an ultrasound-guided diagnostic C2 block, and only those with a positive response were included.

#### 2.1.1 Ethical considerations and consent

This study was approved by the Institutional Review Board of Yanbian University Hospital (No. 2023264) and conformed to the Declaration of Helsinki. Because de-identified retrospective data were used, research-specific informed consent was waived. Written consent for clinical procedures (C2 dorsal root ganglion pulsed radiofrequency and suboccipital myofascial plane block) was obtained from all subjects as part of routine care.

### 2.2 Exclusion criteria

Exclusion criteria included:

(1) Primary headache disorders (migraine, tension-type headache, cluster headache, or trigeminal autonomic cephalalgias);
(2) Cervical disease requiring surgery or magnetic resonance imaging evidence of moderate-to-severe foraminal stenosis;
(3) History of cervical surgery or neurodestructive procedures, coagulopathy (international normalized ratio INR > 1.5), immunodeficiency, or uncontrolled psychiatric disorders.

### 2.3 Study design

This was a single-center, retrospective, controlled cohort study comparing C2 dorsal root ganglion pulsed radiofrequency (C2 DRG PRF) alone (PRF group) vs. PRF combined with an ultrasound-guided suboccipital myofascial plane block (PRF + SMPB group). The prespecified primary endpoint was the between-group difference in visual analog scale at 3 months; secondary endpoints included visual analog scale at 1 week/1 month/6 months; the Short-Form McGill Pain Questionnaire; the Neck Disability Index; monthly attack frequency, and attack duration. Follow-up time points were indexed to PRF as time zero.

Baseline comparability was assessed using the standardized mean difference (SMD). Variables with |SMD| ≥ 0.10 and clinically relevant covariates—including body mass index (BMI), sex, non-steroidal anti-inflammatory drugs (NSAID) use, disease duration, baseline visual analog scale (VAS), Neck Disability Index (NDI), the Short-Form McGill Pain Questionnaire (SF-MPQ) scores, and prior treatments—were prespecified for covariate adjustment. Statistical methodology is detailed in Section 2.7.

### 2.4 Treatment procedures

#### 2.4.1 Ultrasound-Guided C2 DRG PRF (PRF group)

**Figure 1:**
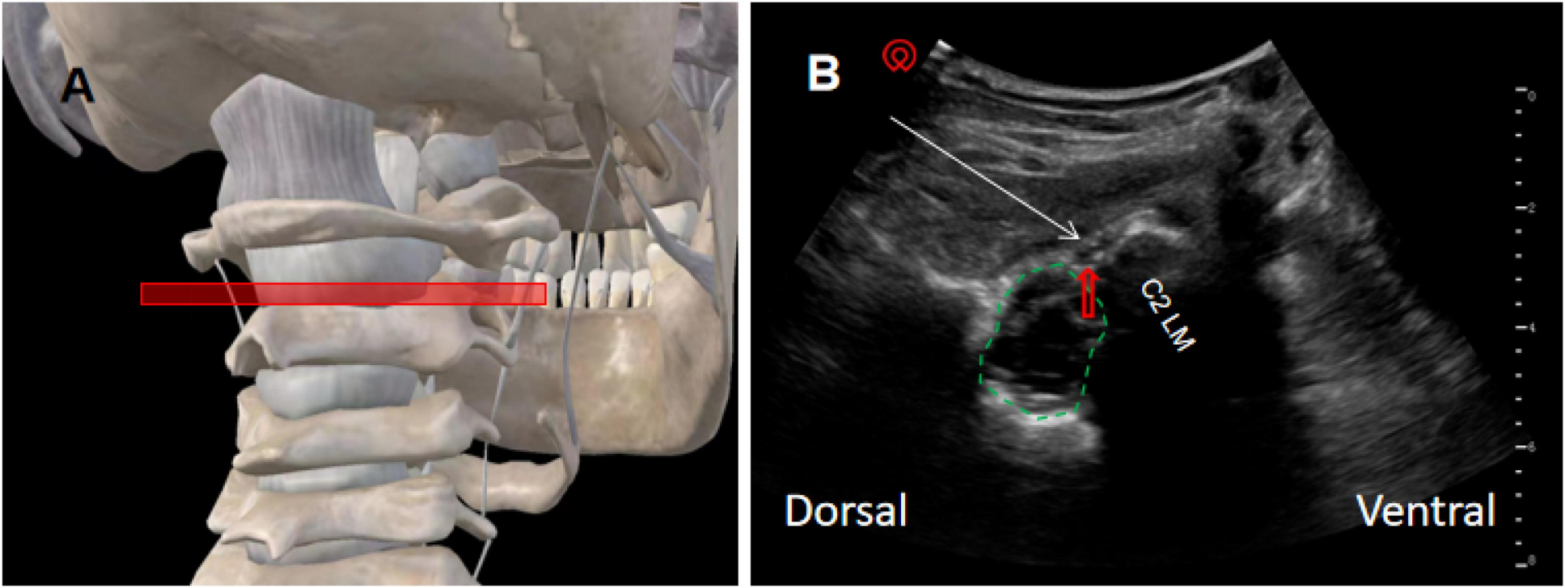
Ultrasound probe positioning and visualization for C2 dorsal root ganglion (DRG) pulsed radiofrequency. **(A)** Anatomical schematic illustrating ultrasound probe placement and orientation for targeting the C2 DRG. **(B)** Corresponding ultrasound image showing needle trajectory (white arrow) toward the C2 DRG (red hollow arrow). The green dashed line outlines the spinal cord, and “C2 LM” denotes the C2 lateral mass. C2 LM, C2 lateral mass.

A 2–5 MHz curvilinear probe (Navi series, Shenzhen, China) was placed transversely between the atlas and axis to visualize the dural sac, spinal cord, C2 DRG, and C2 lateral mass (Figure 1). After local anesthesia (1% lidocaine 3 ml) and in-plane posterior approach, a 22G RF cannula (10 cm, 5-mm active tip) was advanced under ultrasound guidance. Sensory stimulation (50 Hz, ≤0.3 V) confirmed targeting. PRF was delivered using a Cosman G4 generator (Cosman Medical, Burlington, MA, USA) at 42°C, 20-ms pulses, 40–60 V, impedance 150–300 *Ω*, for 600 s. At completion, 0.9% saline 2 ml plus dexamethasone 2 mg was injected. All procedures were performed by a single specialist (>8 years experience).

#### 2.4.2 C2 DRG PRF combined with suboccipital myofascial plane block (PRF + SMPB group)

**Figure 2:**
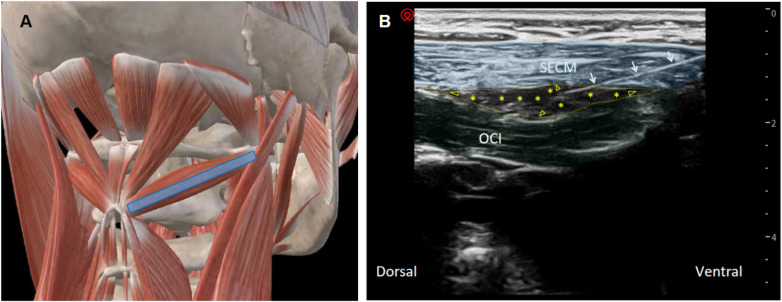
Ultrasound probe positioning and injectate dispersion pattern for the suboccipital myofascial plane block. **(A)** Anatomical schematic illustrating the placement and orientation of the ultrasound probe for accessing the suboccipital fascial plane. **(B)** Corresponding ultrasound image showing the interfascial plane between the semispinalis capitis muscle (SECM, shaded in blue) and the obliquus capitis inferior muscle (OCI, shaded in green). The thin yellow dashed line delineates the fascial boundaries of the target plane. Yellow asterisks indicate the distribution of the injectate within the plane, and yellow hollow arrowheads depict the direction of injectate spread. White arrows demonstrate the needle trajectory. The annotations highlight the anatomical relationships and dispersion pattern without implying muscular boundaries. OCI, obliquus capitis inferior; SECM, semispinalis capitis.

After PRF, a 4–12 MHz linear probe (depth 3–4 cm) was aligned with the long axis of the OCI to identify the fascial plane between OCI and SECM (Figure 2). After local anesthesia, an in-plane puncture delivered 0.5% lidocaine 10 ml + dexamethasone 2.5 mg. SMPB was performed three times (days 0/7/21). Both groups received only one PRF session. All procedures were performed by the same pain specialist.

### 2.5 Clinical assessments

Assessments were performed at baseline and at 1 week, 1 month, 3 months, and 6 months after treatment. The following were collected:

(1) Headache attack frequency: mean number of attacks per month during the assessment interval (recorded at 1/3/6 months).
(2) Headache duration: hours per typical attack (recorded at all time points).
(3) Pain intensity: VAS (0–10; 0 = no pain, 10 = worst pain), averaging the prior week.
(4) SF-MPQ: pain rating index (PRI; 15 items, 0–45) + VAS (0–10) + present pain intensity (PPI; 0–5); total 0–60.
(5) NDI: 10 domains; total 0–50; graded 0–4 (none), 5–14 (mild), 15–24 (moderate), 25–34 (severe), 35–50 (complete).
(6) Treatment-related complications: see Section 2.5.1.

All assessments were conducted by attending physicians using a standardized protocol to ensure consistency and reliability.

#### 2.5.1 Safety monitoring and complications

Predefined, clinically meaningful complications included: intravascular injection or hematoma requiring intervention; local anesthetic systemic toxicity; infection requiring antibiotics; neurological deficits >24 h; systemic corticosteroid reactions requiring treatment; and allergic/anaphylactoid responses. Monitoring included real-time procedural observation, 2-h recovery, 72-h early follow-up, and structured interviews at subsequent visits. No predefined complications nor minor adverse events were recorded.

### 2.6 Treatment allocation and follow-up timing

Treatment allocation considered: (i) severity of myofascial involvement; (ii) response to conservative therapy; and (iii) patient preference. Follow-up timing was standardized to the PRF date.

### 2.7 Statistical analysis

Analyses were performed in SPSS 27.0 using two-sided *α* = 0.05. Baseline balance was evaluated using standardized mean difference (SMD); variables with |SMD| ≥ 0.10 and clinically relevant covariates were included in analysis of covariance (ANCOVA) for the prespecified primary endpoint (3-month VAS). Results are reported as adjusted mean differences (Adjusted Δ) with 95% CI and *p*-values. Hedges' g was reported as effect size.

Secondary outcomes were analyzed using independent-samples *t*-tests (with Welch correction when appropriate). Multiplicity across time points was controlled via the Holm method. Sensitivity analysis using a parsimonious ANCOVA model (age, sex, baseline VAS only) confirmed estimate robustness.

Propensity score approaches (matching or inverse probability weighting) were considered but not implemented due to incomplete baseline individual-level data in this retrospective dataset; therefore, covariate adjustment and SMD assessment were used to control baseline imbalance.

No *a priori* sample size calculation was conducted, as this was a retrospective cohort including all eligible subjects during the study period. However, a *post hoc* power consideration based on the observed between-group difference in the primary endpoint (3-month VAS) indicated that the final sample (*n* = 67) provided >90% statistical power (two-sided *α* = 0.05) to detect a 1-point difference, which is commonly regarded as the minimally clinically important difference in chronic pain outcomes.

Key model assumptions (normality, variance homogeneity, and linearity) were verified using residual plots, Q-Q plots, and Levene's test, with no substantial violations. Analyses were performed on complete cases; missing secondary endpoints were evaluated via available-case analysis.

Safety results were summarized by group using counts and percentages; χ^2^ or Fisher's exact test was applied where appropriate.

## 3 Results

### 3.1 Enrollment and baseline characteristics

**Figure 3:**
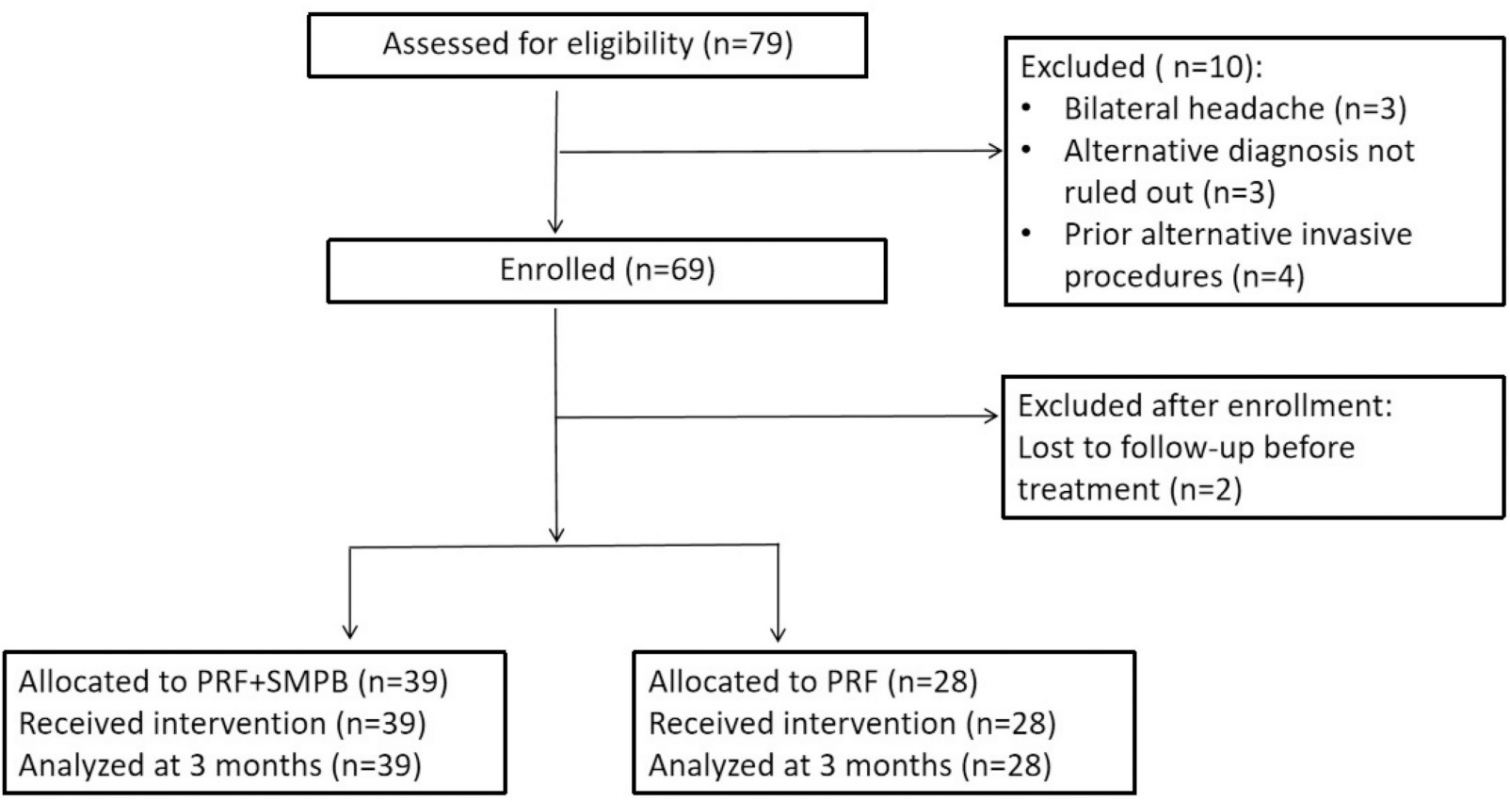
STROBE flow diagram of study enrollment, allocation, and analysis.

A total of 79 subjects were screened; 10 were excluded, 69 were enrolled, and 2 withdrew before the initial treatment, leaving 67 subjects for analysis (PRF + SMPB *n* = 39; PRF *n* = 28; Figure 3). The cohort included 31 men and 36 women, mean age 54.64 ± 9.95 years, body mass index (BMI) 22.72 ± 2.32 kg/m^2^, mean headache duration 24.04 ± 10.51 months, with 36 right- and 31 left-sided cases (Table 1). The primary endpoint (3-month visual analog scale VAS) was available for all 67 subjects.

**Table 1:** Baseline characteristics of the study population by treatment group.

Variable	PRF + SMPB (*n* = 39)	PRF (*n* = 28)	Std. diff., |SMD|^a^
Age, years (mean ± SD)	53.05 ± 9.96	56.86 ± 9.69	0.39
Duration of symptoms, months (mean ± SD)	24.82 ± 11.27	22.96 ± 9.45	0.18
Affected side, *n* (%)			
Right	21 (53.8)	15 (53.6)	0.01^b^
Left	18 (46.2)	13 (46.4)	–
Sex, n (%)			
Male	20 (51.3)	11 (39.3)	0.24^b^
Female	19 (48.7)	17 (60.7)	–
BMI, kg/m^2^ (mean ± SD)	22.41 ± 2.22	23.16 ± 2.42	0.33
Prior treatments, *n* (%)^c^			
NSAIDs	32 (82.1)	19 (67.9)	0.33^b^
Muscle relaxants	6 (15.4)	3 (10.7)	0.14^b^
Manipulative therapy	32 (82.1)	24 (85.7)	0.10^b^
Physiotherapy	20 (51.3)	14 (50.0)	0.03^b^
Topical plasters/patches	23 (59.0)	14 (50.0)	0.18^b^
Oral Chinese medicine	23 (59.0)	15 (53.6)	0.11^b^

Percentages are column-wise and may not total 100% due to rounding. PRF, pulsed radiofrequency of the C2 dorsal root ganglion; SMPB, suboccipital myofascial plane block; BMI, body mass index; SMD, standardized mean difference.

a Standardized mean differences (SMDs) are reported as absolute values. For continuous variables: SMD = (mean₁−mean₂)/pooled SD. For binary variables: SMD = (p₁−p₂)/√{p₁(1−p₁)+p₂(1−p₂)/2}. Thresholds: <0.10 negligible; 0.10–0.20 small; 0.20–0.50 moderate.

b For multi-level categorical variables, SMD is shown for one level (e.g., Right for affected side; Male for sex).

c Prior treatments were recorded within the predefined look-back window and were not mutually exclusive (multiple selections allowed).

Baseline balance was evaluated using standardized mean differences (SMDs). Mild–moderate imbalance was noted for age (≈0.39), BMI (≈0.33), male sex (≈0.24), and nonsteroidal anti-inflammatory drug (NSAID) use (≈0.33); therefore, these were prespecified as covariates in the adjusted primary endpoint analysis (Section 2.7).

### 3.2 Pain intensity (VAS, 0–10)

**Figure 4:**
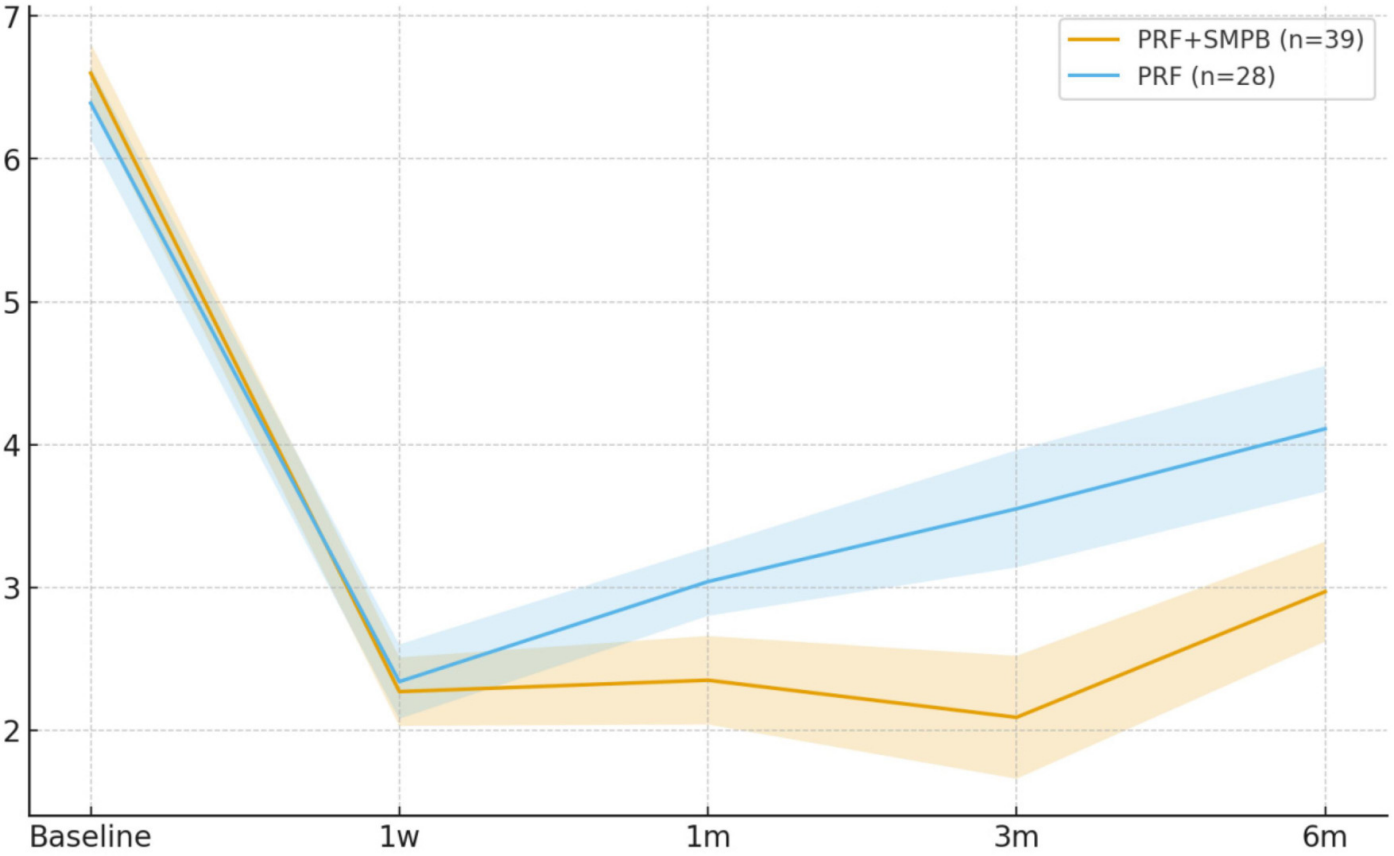
Temporal trend of pain intensity (VAS, 0–10) in the PRF + SMPB group and PRF-alone group from baseline to 6 months post-treatment. Error bars represent 95% confidence intervals.

VAS decreased significantly from baseline in both groups at all post-treatment time points (Table 2). Between-group comparisons showed a sustained advantage for PRF + SMPB beginning at 1 month: 1 month Δ = −0.69 (95% CI −1.08, −0.30; *p* = 0.002), 3 months (primary endpoint) Δ = −1.46 (95% CI −2.06, −0.86; *p* < 0.001; Hedges' g = −1.13), and 6 months Δ = −1.14 (95% CI −1.70, −0.58; *p* < 0.001; g = −0.98). No significant difference was seen at 1 week (Δ = −0.07; 95% CI −0.42, +0.28; *p* = 0.701). ANCOVA-adjusted estimates are summarized in Table 3. Longitudinal VAS progression is shown in Figure 4.

**Table 2:** Comparison of pain intensity (VAS, 0–10) between groups at each time point.

Time point	PRF + SMPB (*n* = 39), mean ± SD	95% CI	PRF (*n* = 28), mean ± SD	95% CI	Between-group difference Δ (95% CI)^a^	Hedges’ g	*t*	*p*
Baseline	6.60 ± 0.67	6.39–6.81	6.39 ± 0.67	6.14–6.64	+0.21 (−0.12, +0.54)	+0.31	1.262	0.212
1 week	2.27 ± 0.75*	2.03–2.51	2.34 ± 0.71*	2.08–2.60	−0.07 (−0.42, +0.28)	−0.09	−0.386	0.701
1 month	2.35 ± 0.99*	2.04–2.66	3.04 ± 0.65*	2.80–3.28	−0.69 (−1.08, −0.30)	−0.79	−3.221	0.002
3 months^b^	2.09 ± 1.38*	1.66–2.52	3.55 ± 1.11*	3.14–3.96	−1.46 (−2.06, −0.86)	−1.13	−4.774	<0.001
6 months	2.97 ± 1.11*	2.62–3.32	4.11 ± 1.20*	3.67–4.55	−1.14 (−1.70, −0.58)	−0.98	−3.984	<0.001

*p*-values represent Holm-adjusted results for multiplicity control across post-treatment timepoints.

VAS, visual analog scale; PRF, pulsed radiofrequency of the C2 dorsal root ganglion; SMPB, suboccipital myofascial plane block; CI, confidence interval.

a Is defined as *(PRF + SMPB)−(PRF)*; negative values favor PRF + SMPB (lower VAS).

95% CIs were derived using Welch's unequal-variance two-sample *t*-test.

Group comparisons used independent-samples *t*-tests; effect size reported as Hedges’ *g* (small ≈ 0.2, medium ≈ 0.5, large ≥0.8).

b Primary endpoint.

* Significant within-group change from baseline, *p* < 0.05.

**Table 3:** ANCOVA-adjusted estimates for VAS outcomes (primary endpoint).

Timepoint	Adjusted mean difference Δ (95% CI)	*p*-value
1 week	−0.07 (−0.42, +0.28)	0.701
1 month	−0.69 (−1.08, −0.30)	0.002
3 months^a^	−1.46 (−2.06, −0.86)	<0.001
6 months	−1.14 (−1.70, −0.58)	<0.001

Δ is defined as (PRF + SMPB)−(PRF); negative values favor PRF + SMPB.

Adjusted values derived from ANCOVA controlling for baseline VAS and imbalanced covariates.

Holm-adjusted *p*-values are reported for comparisons across time points.

VAS, Visual Analog Scale; CI, confidence interval.

a Prespecified primary endpoint.

### 3.3 Headache attack frequency (episodes per month)

**Figure 5:**
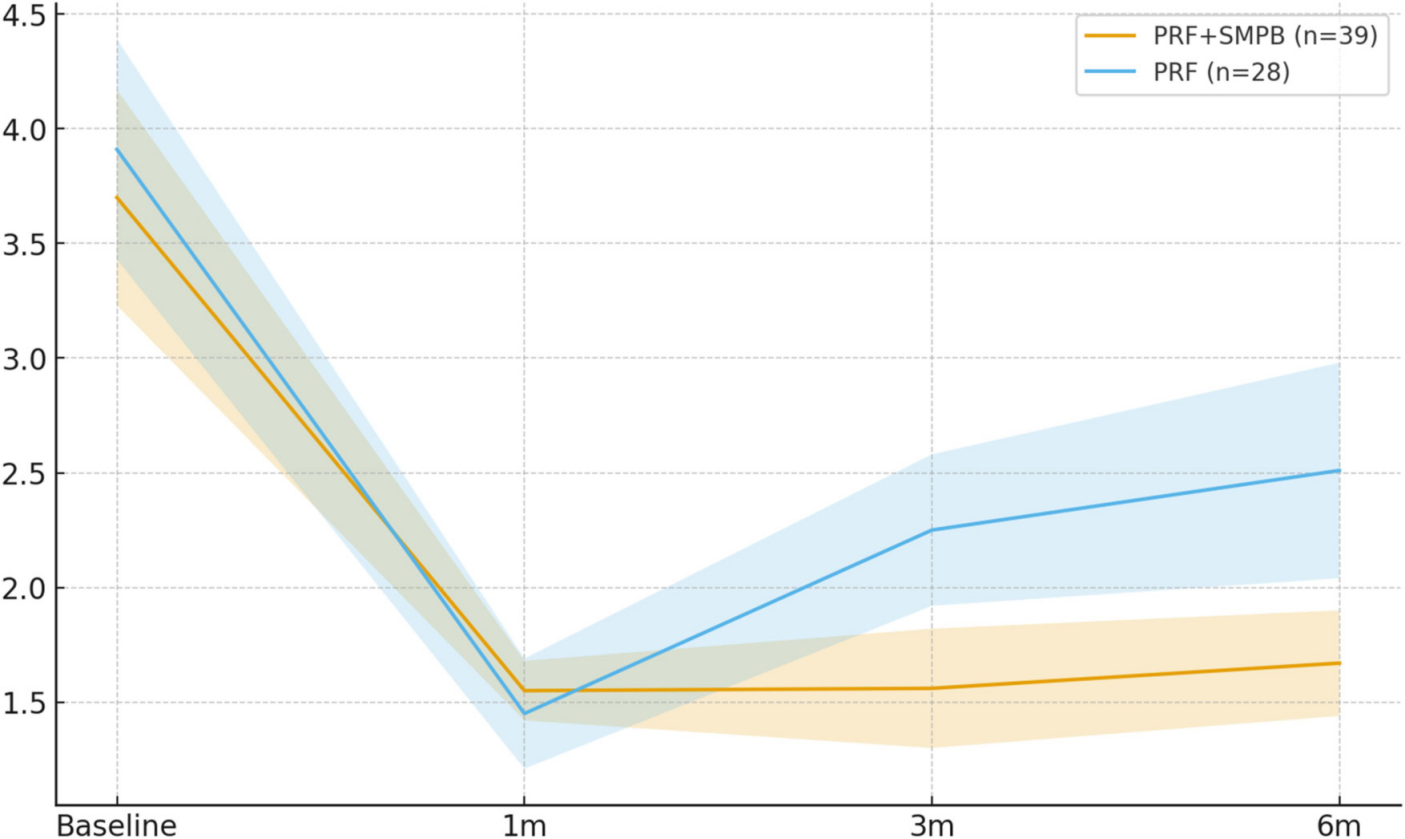
Temporal trend of headache attack frequency (episodes per month) in the PRF + SMPB group and PRF group from baseline to 6 months post-treatment. Error bars represent 95% confidence intervals.

Monthly assessments demonstrated significantly fewer attacks with PRF + SMPB at 3 months (Δ = −0.69; 95% CI −1.10, −0.28; *p* = 0.001; g = −0.84) and 6 months (Δ = −0.84; 95% CI −1.34, −0.34; *p* = 0.001; g = −0.88). The 1-month difference was nonsignificant (Δ = +0.10; 95% CI −0.17, +0.37; *p* = 0.470; Table 4). Figure 5 illustrates the temporal reduction.

**Table 4:** Headache attack frequency (episodes per month) by group.

Time point	PRF + SMPB (*n* = 39), mean ± SD	95% CI	PRF (*n* = 28), mean ± SD	95% CI	Between-group difference Δ (95% CI)^a^	Hedges’ g	*t*	*p*
Baseline	3.70 ± 1.41	3.26–4.14	3.91 ± 1.80	3.24–4.58	−0.21 (−1.01, 0.59)	−0.13	−0.491	0.624
1 month	1.55 ± 0.39*	1.43–1.67	1.45 ± 0.65*	1.21–1.69	+0.10 (−0.17, 0.37)	+0.19	0.726	0.470
3 months	1.56 ± 0.74*	1.33–1.79	2.25 ± 0.91*	1.91–2.59	−0.69 (−1.10, −0.28)	−0.84	−3.305	0.001
6 months	1.67 ± 0.71*	1.45–1.89	2.51 ± 1.20*	2.07–2.95	−0.84 (−1.34, −0.34)	−0.88	−3.312	0.001

*p*-values represent Holm-adjusted results for multiplicity control across post-treatment timepoints.

If multiplicity adjustment across post-treatment time points is required, Holm-adjusted *q*-values are: 1 month = 0.470, 3 months = 0.003, 6 months = 0.003.

PRF, pulsed radiofrequency of the C2 dorsal root ganglion; SMPB, suboccipital myofascial plane block; CI, confidence interval.

a Δ is defined as *(PRF + SMPB)−(PRF)*; negative values favor PRF + SMPB (fewer episodes).

95% confidence intervals (CIs) were derived using Welch's unequal-variance two-sample *t*-test.

Group comparisons used independent-samples *t*-tests; effect size reported as Hedges’ *g* (small ≈ 0.2, medium ≈ 0.5, large ≥0.8).

* Significant within-group change from baseline (*paired* *t*-test), *p* < 0.05.

### 3.4 Duration per attack (hours/episode)

**Figure 6:**
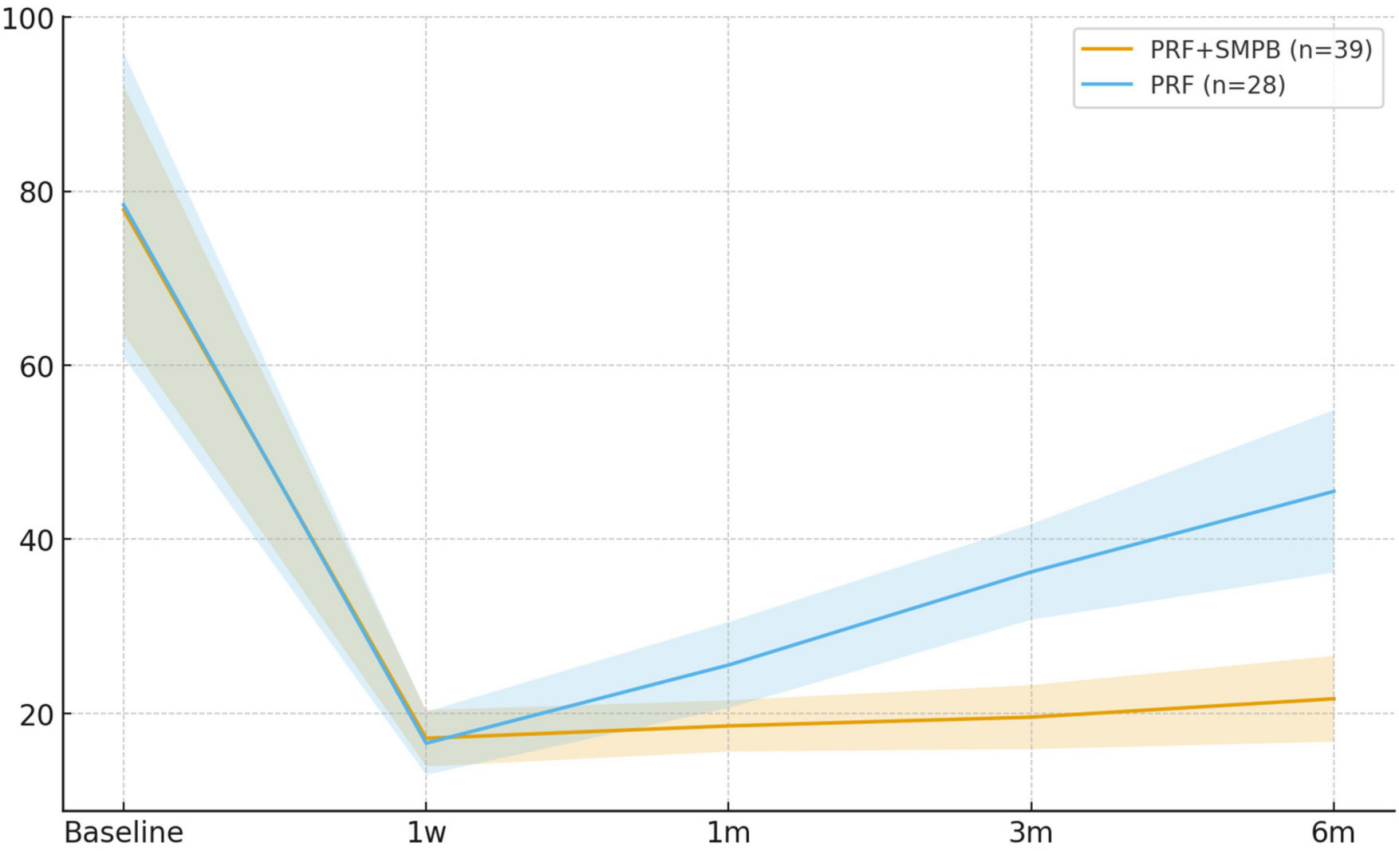
Temporal trend of headache attack duration (hours per episode) in the PRF + SMPB group and PRF group from baseline to 6 months post-treatment. Error bars represent 95% confidence intervals.

PRF + SMPB resulted in shorter attack duration at 1 month (Δ = −7.00; 95% CI −12.76, −1.24; *p* = 0.020), 3 months (Δ = −16.69; 95% CI −23.30, −10.08; *p* < 0.001), and 6 months (Δ = −23.84; 95% CI −34.40, −13.28; *p* < 0.001) (Table 5). No significant difference occurred at 1 week (Δ = +0.57; 95% CI −4.29, +5.43; *p* = 0.818). Figure 6 presents the longitudinal change.

**Table 5:** Headache attack duration (hours per episode) by group.

Time point	PRF + SMPB (*n* = 39), mean ± SD	95% CI	PRF (*n* = 28), mean ± SD	95% CI	Between-group difference Δ (95% CI)^a^	Hedges’ g	*t*	*p*
Baseline	77.85 ± 45.62	63.53–92.17	78.44 ± 47.25	60.94–95.94	−0.59 (−23.20, +22.02)	−0.01	−0.051	0.959
1 week	17.13 ± 10.33*	13.89–20.37	16.56 ± 9.76*	12.94–20.18	+0.57 (−4.29, +5.43)	+0.06	0.230	0.818
1 month	18.55 ± 9.39*	15.60–21.50	25.55 ± 13.35*	20.61–30.49	−7.00 (−12.76, −1.24)	−0.62	−2.380	0.020
3 months	19.56 ± 11.74*	15.88–23.24	36.25 ± 14.81*	30.76–41.74	−16.69 (−23.30, −10.08)	−1.26	−4.950	<0.001
6 months	21.67 ± 15.71*	16.74–26.60	45.51 ± 25.20*	36.18–54.84	−23.84 (−34.40, −13.28)	−1.17	−4.426	<0.001

*p*-values represent Holm-adjusted results for multiplicity control across post-treatment timepoints.

PRF, pulsed radiofrequency of the C2 dorsal root ganglion; SMPB, suboccipital myofascial plane block; CI, confidence interval.

a Δ is defined as *(PRF + SMPB)−(PRF)*; negative values favor PRF + SMPB (shorter duration).

95% confidence intervals (CIs) were derived from two-sample standard errors (Welch approximation).

Group comparisons used independent-samples *t*-tests; effect size reported as Hedges’ *g* (small ≈ 0.2, medium ≈ 0.5, large ≥0.8).

* Significant within-group change from baseline (*paired* *t*-test), *p* < 0.05.

### 3.5 Short-form McGill pain questionnaire (SF-MPQ, 0–60)

**Figure 7:**
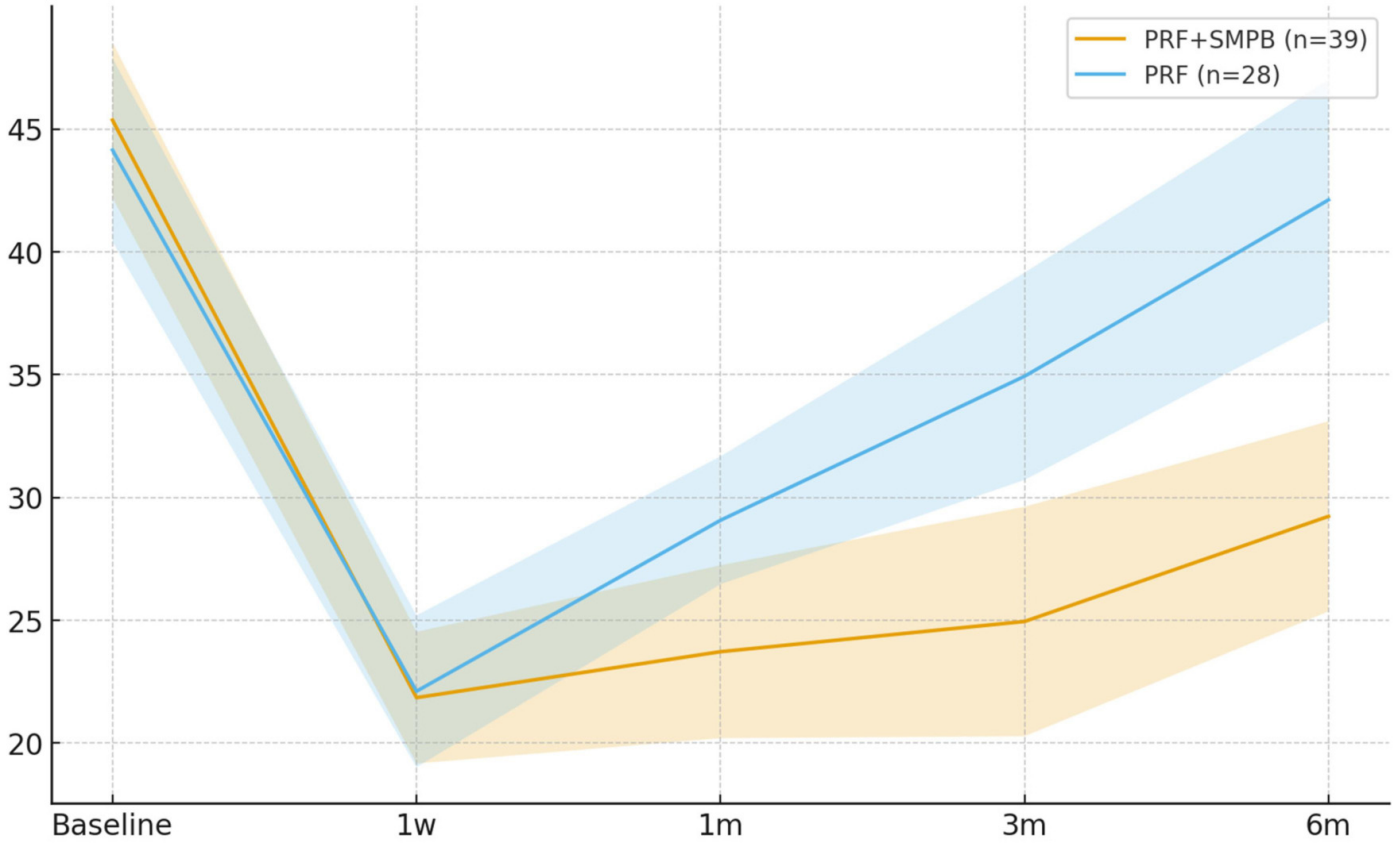
Temporal trend of SF-MPQ total score in the PRF + SMPB group and PRF-only group from baseline to 6 months post-treatment. Error bars represent 95% confidence intervals.

Significantly lower SF-MPQ total scores were observed in the PRF + SMPB group at 1 month (Δ = −5.35; 95% CI −9.67, −1.03; *p* = 0.016), 3 months (Δ = −9.98; 95% CI −16.31, −3.65; *p* = 0.002), and 6 months (Δ = −12.88; 95% CI −19.20, −6.56; *p* < 0.001) (Table 6). The 1-week difference was nonsignificant (Δ = −0.26; 95% CI −4.04, +3.52; *p* = 0.891). Time-course changes appear in Figure 7.

**Table 6:** SF-MPQ total score (0–60) by group at each time point.

Time point	PRF + SMPB (*n* = 39), mean ± SD	95% CI	PRF (*n* = 28), mean ± SD	95% CI	Between-group difference Δ (95% CI)^a^	Hedges’ g	*t*	*p*
Baseline	45.36 ± 9.82	42.28–48.44	44.14 ± 9.91	40.47–47.81	+1.22 (−3.68, +6.12)	+0.12	0.499	0.620
1 week	21.85 ± 8.35*	19.23–24.47	22.11 ± 7.09*	19.48–24.74	−0.26 (−4.04, +3.52)	−0.03	−0.137	0.891
1 month	23.72 ± 10.75*	20.35–27.09	29.07 ± 6.91*	26.51–31.63	−5.35 (−9.67, −1.03)	−0.57	−2.476	0.016
3 months	24.95 ± 14.76*	20.32–29.58	34.93 ± 11.17*	30.79–39.07	−9.98 (−16.31, −3.65)	−0.74	−3.149	0.002
6 months	29.23 ± 12.35*	25.35–33.11	42.11 ± 13.00*	37.29–46.93	−12.88 (−19.20, −6.56)	−1.01	−4.084	<0.001

*p*-values represent Holm-adjusted results for multiplicity control across post-treatment timepoints.

95% confidence intervals (CIs) were derived using Welch's unequal-variance two-sample *t*-test.

Group comparisons used independent-samples *t*-tests; effect size reported as Hedges’ *g* (small ≈ 0.2, medium ≈ 0.5, large ≥0.8).

SF-MPQ, Short-Form McGill Pain Questionnaire; PRF, pulsed radiofrequency of the C2 dorsal root ganglion; SMPB, suboccipital myofascial plane block; CI, confidence interval.

a Δ is defined as *(PRF + SMPB)−(PRF)*; negative values favor PRF + SMPB (lower SF-MPQ total score).

* Significant within-group change from baseline (*paired* *t*-test), *p* < 0.05.

### 3.6 Neck disability index (NDI, 0–50)

**Figure 8:**
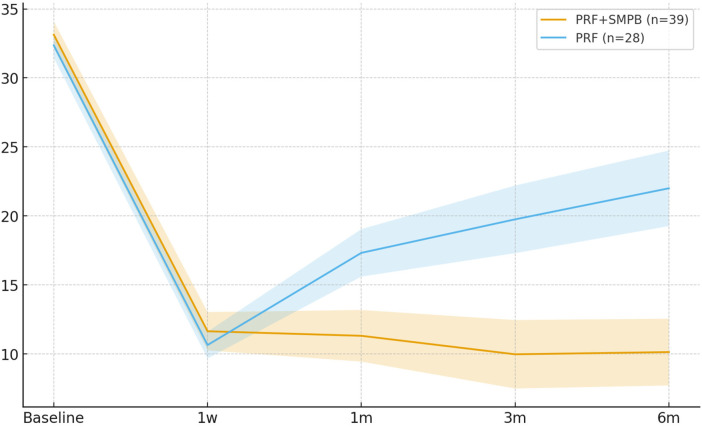
Temporal trend of the neck disability Index (NDI, 0–50) in the PRF + SMPB and PRF groups from baseline to 6 months post-treatment. Error bars represent 95% confidence intervals.

Functional improvement was greater with PRF + SMPB at 1 month (Δ = −6.01; 95% CI −8.50, −3.52; *p* < 0.001), 3 months (Δ = −9.78; 95% CI −13.16, −6.40; *p* < 0.001), and 6 months (Δ = −11.87; 95% CI −15.41, −8.33; *p* < 0.001) (Table 7). At 1 week, the difference was not significant (Δ = +1.00; 95% CI −0.66, +2.66; *p* = 0.283). Figure 8 depicts the improvement trajectory.

**Table 7:** Neck disability Index (NDI, 0–50) by group at each time point.

Time point	PRF + SMPB (*n* = 39), mean ± SD	95% CI	PRF (*n* = 28), mean ± SD	95% CI	Between-group difference Δ (95% CI)^a^	Hedges’ g	*t*	*p*
Baseline	33.13 ± 2.98	32.17–34.09	32.36 ± 2.38	31.45–33.27	+0.77 (−0.52, +2.06)	+0.28	1.135	0.260
1 week	11.64 ± 4.39*	10.25–13.03	10.64 ± 2.51*	9.67–11.61	+1.00 (−0.66, +2.66)	+0.27	1.082	0.283
1 month	11.31 ± 5.81*	9.44–13.18	17.32 ± 4.56*	15.60–19.04	−6.01 (−8.50, −3.52)	−1.12	−4.560	<0.001
3 months	9.97 ± 7.71*	7.49–12.45	19.75 ± 6.38*	17.30–22.20	−9.78 (−13.16, −6.40)	−1.34	−5.591	<0.001
6 months	10.13 ± 7.52*	7.71–12.55	22.00 ± 7.13*	19.26–24.74	−11.87 (−15.41, −8.33)	−1.59	−6.511	<0.001

*p*-values represent Holm-adjusted results for multiplicity control across post-treatment timepoints.

95% confidence intervals (CIs) were derived using Welch's unequal-variance two-sample *t*-test.

Group comparisons used independent-samples *t*-tests; effect size reported as Hedges’ *g* (small ≈ 0.2, medium ≈ 0.5, large ≥0.8).

NDI, neck disability index; PRF, pulsed radiofrequency of the C2 dorsal root ganglion; SMPB, suboccipital myofascial plane block; CI, confidence interval.

a Δ is defined as *(PRF + SMPB)−(PRF)*; negative values favor PRF + SMPB (lower NDI indicates less disability).

* Significant within-group change from baseline (*paired* *t*-test), *p* < 0.05.

### 3.7 Safety

No prespecified complications—namely intravascular injection/hematoma requiring intervention, local anesthetic systemic toxicity, infection requiring antibiotics, neurologic abnormalities >24 h, systemic corticosteroid reactions requiring treatment, or allergic/anaphylactoid responses—were observed in either group during the 6-month follow-up (0 events, 0%). All subjects completed follow-up (attrition 0%). No minor adverse events were reported.

## 4 Discussion

### 4.1 Key findings

This study shows that, compared with ultrasound-guided C2 dorsal root ganglion pulsed radiofrequency (C2 DRG PRF) alone, C2 DRG PRF combined with suboccipital myofascial plane block (PRF + SMPB) yields superior long-term pain relief and functional improvement in cervicogenic headache (CEH). Both groups exhibit significant reductions in visual analog scale (VAS), Short-Form McGill Pain Questionnaire (SF-MPQ), and Neck Disability Index (NDI) scores at 1 week (*p* < 0.05), whereas the advantage of PRF + SMPB emerges at 1 month, becomes more pronounced at 3 months, and is maintained at 6 months (*p* < 0.05). These findings suggest that suboccipital myofascial plane block (SMPB), via a mechanism distinct from pure neuromodulation, enhances and prolongs the therapeutic effect of PRF.

### 4.2 Mechanistic interpretation of short- and long-term efficacy

From an anatomical and pathophysiological perspective, CEH arises from the convergence of neural sensitization and persistent myofascial nociceptive input (6, 7). Degenerative or pathological changes involving the atlantoaxial joints and upper cervical segments generate abnormal afferent signaling from C1–C3 spinal nerves, facilitating central sensitization within the trigeminocervical complex (8–10). In parallel, dysfunction of cervical soft tissues—including muscle injury, spasm, and myofascial inflammatory responses—provides sustained peripheral nociceptive drive and may mechanically or chemically irritate the occipital nerves traversing these structures, thereby contributing to headache generation and maintenance (10–12). This dual-pathway model provides a biological rationale for therapeutic strategies that simultaneously target both central neural modulation and peripheral myofascial mechanisms.

Under ultrasound guidance, C2 dorsal root ganglion pulsed radiofrequency (C2 DRG PRF) modulates nociceptive transmission through exposure to pulsed electric fields, attenuating abnormal afferent input and central sensitization without causing neural destruction (15, 17–20). The ultrasound-guided posterior approach used in this study enables clear visualization of the C2 DRG, spinal cord, and dural sac within the atlantoaxial interval, offering procedural safety and targeting precision comparable to previously reported techniques by Wu et al. (19) and Hua et al. (20). Although PRF alone produced significant short-term symptom relief, the relatively modest durability of its effect in some subjects underscores the multifactorial nature of CEH and suggests that neuromodulation alone may be insufficient when substantial myofascial pathology persists.

Suboccipital myofascial plane block (SMPB) addresses this complementary peripheral component. Ultrasound-guided myofascial plane blocks are thought to alleviate chronic myofascial pain through accurate intrafascial drug deposition, mechanical release of fascial adhesions, and attenuation of aseptic inflammatory processes that perpetuate nociceptive sensitization (26, 30, 34, 35). In the present study, dexamethasone was administered both following PRF and within SMPB injectates. As a potent glucocorticoid, dexamethasone suppresses pro-inflammatory cytokine activity and enhances sustained analgesia (31). Clinical trials and meta-analyses further indicate that perineural dexamethasone prolongs block duration and reduces neuritis and tissue edema without compromising safety (32, 33, 39, 40). When combined with hydrodissection, SMPB may reduce inflammatory burden surrounding the C2 DRG as well as the greater and third occipital nerves (GON/TON), thereby diminishing ongoing peripheral nociceptive input; however, these mechanistic effects warrant confirmation in prospective studies.

The temporal pattern observed in this cohort—minimal between-group differences at 1 week followed by progressively greater separation at 1, 3, and 6 months—supports a cumulative and synergistic interaction between PRF and SMPB rather than a purely immediate analgesic effect. PRF likely provides early neuromodulatory stabilization of central pain processing, whereas repeated SMPB sessions promote gradual peripheral desensitization and restoration of myofascial tissue function. This interpretation is consistent with prior evidence indicating that serial greater occipital nerve blocks yield cumulative benefit in occipital-related headache disorders (36, 37), and that repeated fascial interventions are often required to achieve durable improvement in myofascial pain syndromes (26). Meta-analytic data further support dose- and time-dependent effects of perineural dexamethasone (38–40). Accordingly, the three-session SMPB protocol applied within 21 days in this study reflects pragmatic clinical practice, although optimal treatment frequency remains to be determined.

Cadaveric investigations provide additional anatomical support for this combined approach. Injectate placed within the fascial plane between the obliquus capitis inferior (OCI) and semispinalis capitis muscle (SECM) consistently spreads to the GON and TON (22), and compression or irritation originating from these muscles has been implicated in occipital pain generation (24, 25, 41). By translating these anatomical insights into a targeted clinical intervention, SMPB complements C2 DRG PRF by addressing both neural and myofascial contributors to CEH. The consistent improvements observed across pain intensity, attack frequency and duration, SF-MPQ, and NDI—peaking at 3 months and persisting through 6 months—are therefore more plausibly explained by synergistic neuromodulation and progressive peripheral desensitization than by statistical variation alone.

### 4.3 Limitations

This study has limitations. First, as a retrospective, non-randomized observational cohort, causality cannot be established. Treatment allocation was influenced by clinical severity and patient preference, exposing results to confounding by indication and unmeasured variables such as emotional status, rehabilitation adherence, home-based exercise, and concurrent therapies. Although baseline imbalance was adjusted using analysis of covariance (ANCOVA) based on standardized mean difference (SMD) assessment, residual confounding cannot be excluded. Second, treatment exposure was asymmetric, with three SMPB sessions in the PRF + SMPB group vs. a single PRF in the comparison group, which may have amplified group differences and limits attribution of independent effects. Third, propensity score-based adjustment could not be applied due to incomplete individual-level baseline data; no *a priori* sample size estimation was performed, and results should be viewed as exploratory. Fourth, secondary outcomes used complete-case/available-case analysis, and 6-month follow-up may be insufficient to assess long-term durability. Fifth, rehabilitation and other adjunctive treatments, known to influence CEH outcomes (3, 13), were not systematically collected. Finally, the single-center design and modest sample size may limit generalizability. Prospective multicenter randomized controlled trials with standardized exposure, predefined sample size, structured rehabilitation monitoring, and longer follow-up are needed to validate these preliminary findings and optimize treatment schedules.

## 5 Conclusions

In this retrospective cohort of 67 subjects with unilateral cervicogenic headache (CEH), ultrasound-guided C2 dorsal root ganglion pulsed radiofrequency (C2 DRG PRF) combined with suboccipital myofascial plane block (SMPB) provided significantly greater and more sustained improvements in visual analog scale (VAS), Neck Disability Index (NDI) and the Short-Form McGill Pain Questionnaire (SF-MPQ) scores at 1, 3, and 6 months compared with pulsed radiofrequency (PRF) alone, indicating superior pain relief and functional recovery.

However, these findings must be interpreted cautiously due to the retrospective, non-randomized design, unequal treatment exposure, small sample size, and incomplete data on rehabilitation and concomitant therapies. Thus, the results remain exploratory and hypothesis-generating rather than confirmatory.

Future prospective randomized controlled trials with standardized treatment cycles and longer follow-up are needed to validate these preliminary observations and determine the independent contribution of the combined intervention.

## Data Availability

The raw data supporting the conclusions of this article will be made available by the authors, without undue reservation.
